# Recurrent aneurysm 11 years after thoracoabdominal aortic replacement due to recanalization of intercostal arteries of the wrapping native aorta in Marfan syndrome: a case report

**DOI:** 10.1186/s44215-023-00084-9

**Published:** 2023-08-03

**Authors:** Shun Tanaka, Haruo Yamauchi, Sachito Minegishi, Minoru Ono

**Affiliations:** Department of Cardiac Surgery, The University of Tokyo, 7-3-1, Hongo, Bunkyo-Ku, Tokyo 113-8655 Japan

**Keywords:** Aortic aneurysm, Surgery, Type II endoleak, Anticoagulation

## Abstract

**Background:**

Persistent blood flow is common in aortic aneurysms following endovascular repair. However, the recanalization of thrombosed intercostal arteries after open aortic repair is rare. We report a case of aortic aneurysm recurrence 11 years after thoracoabdominal aortic replacement resulting from recanalization of the intercostal arteries like a type II endoleak in a patient with Marfan syndrome.

**Case presentation:**

A 50-year-old woman with Marfan syndrome presented with left-sided back pain after meals. She underwent abdominal aortic replacement at 35 years of age, distal aortic arch and thoracoabdominal aortic replacement for type B chronic aortic dissection at 38 years of age, and aortic root to the remnant arch replacement with a mechanical valve conduit for type A acute aortic dissection at 43 years of age, which resulted in complete replacement of the entire aorta with prosthetic grafts. The patient has been taking warfarin since then. The prothrombin time-international normalized ratio levels were controlled almost within the range of 1.5–2.5 during the follow-up period, but it was elevated up to 3.4 at 1 month before she felt back pain and kept high at 4.2 on admission. Enhanced computed tomography (CT) revealed an aneurysm between the descending graft and the remaining native aortic wall, compressing the esophagus. However, there were no abnormal findings on the CT performed 10 months prior to admission. Surgery revealed that the orifices of the intercostal arteries were patent with backflow. We closed the patent intercostal arteries using nonabsorbable sutures. The patient’s postoperative course was uneventful, and the CT performed 1 year later showed no aneurysmal recurrence.

**Conclusions:**

Here, we describe a rare case of recurrent aortic aneurysm long term after graft replacement due to recanalization of the intercostal arteries of the remaining aortic wall. Our findings suggest that closing all orifices of the intercostal arteries during aortic repair, regardless of backflow, could prevent delayed reopening; systemic anticoagulation after aortic replacement may be a risk factor for recanalization, and that postoperative periodic CT follow-up is essential, especially in patients receiving anticoagulant therapy.

## Background

Persistent reverse blood flow from the intercostal and lumbar arteries into the aortic aneurysm after an endovascular repair is common and is known as a type II endoleak. However, recanalization of thrombosed intercostal arteries after an open aortic repair is rare [1, 2]. Herein, we report a case of induced aortic aneurysm recurrence after thoracoabdominal aortic replacement in a patient with Marfan syndrome. We describe our detailed clinical findings, treatment, and outcomes of this case.

## Case presentation

**Fig. 1:**
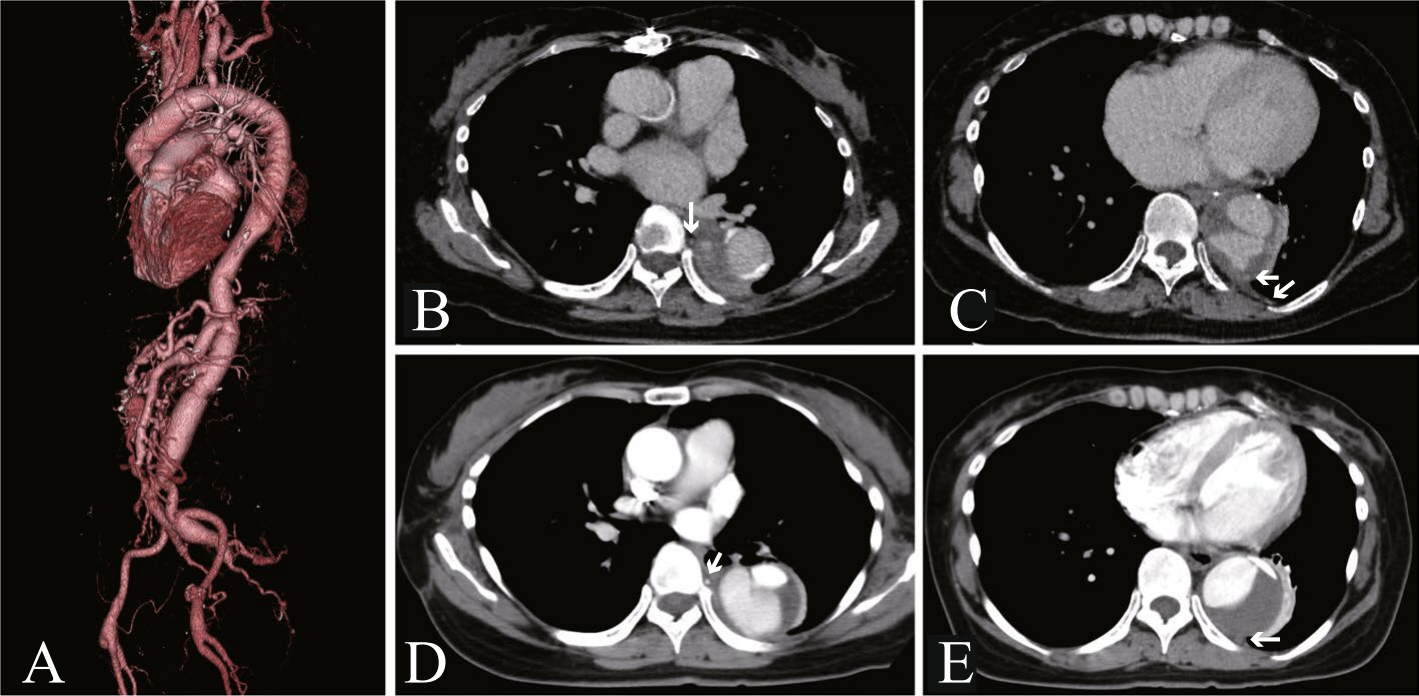
Computed tomography images of the aneurysm. **A** A 3D computed tomography (CT) image of the whole aorta. **B** and **C** CT images showing the recurrent aneurysm. Two contrast extravasation sites detected in the venous phase were distant from the anastomotic site and linked to the right eighth (**B**) and left tenth (**C**) intercostal arteries, indicated by white arrows. **D** and **E** CT images before thoracoabdominal aortic replacement revealing that all intercostal arteries originated from the false lumen; the right eighth intercostal artery was open (**D**), while the left tenth was occluded by a thrombus in the false lumen (**E**). The right eighth and left tenth intercostal arteries are indicated by white arrows

A 50-year-old woman diagnosed with Marfan syndrome presented with a 1-month history of left-sided back pain after meals. She did not undergo genetic testing but was diagnosed with Marfan syndrome based on her family history and clinical manifestations according to the revised Ghent criteria. The aorta was completely replaced in stages with prosthetic grafts (Fig. 1A). The patient had undergone abdominal aortic aneurysm replacement at 35 years of age and developed a type B acute aortic dissection 1 year later. When she was 38 years of age, the distal aortic arch and thoracoabdominal aorta were separately replaced with prosthetic grafts. At 43 years of age, she developed a type A acute aortic dissection and underwent replacement of the aortic root and the remaining aortic arch with a mechanical valved conduit. She has been taking warfarin ever since. The prothrombin time-international normalized ratio (PT-INR) was controlled almost within 1.5–2.5 during the follow-up period. It was elevated up to 3.4 at 1 month before she felt back pain. However, she did not experience any sign of ill-health, such as a common cold or gastroenteritis, which could affect the PT-INR. When the patient visited our department complaining of left back pain, her PT-INR level was 4.2. Enhanced computed tomography (CT) showed an aneurysm (55 mm) around the descending aortic graft compressing the esophagus. However, there were no abnormal findings on CT performed 10 months earlier. Aneurysmal expansion was considered caused by a hematoma between the graft and the remaining native aortic wall, which was used to wrap the graft. No contrast extravasation was evident in the arterial phase; however, there were two sites of mild extravasation in the venous phase (Fig. 1B, C). We suspected that the aneurysm recurred due to the backflow of blood from the right eighth and left tenth intercostal arteries of the wrapped aortic wall rather than anastomotic leakage. CT performed before the thoracoabdominal replacement showed that all intercostal arteries originated from the false lumen. The right 8th intercostal artery was patent (Fig. 1D), whereas the left 10th artery was occluded by a thrombus (Fig. 1E).

**Fig. 2:**
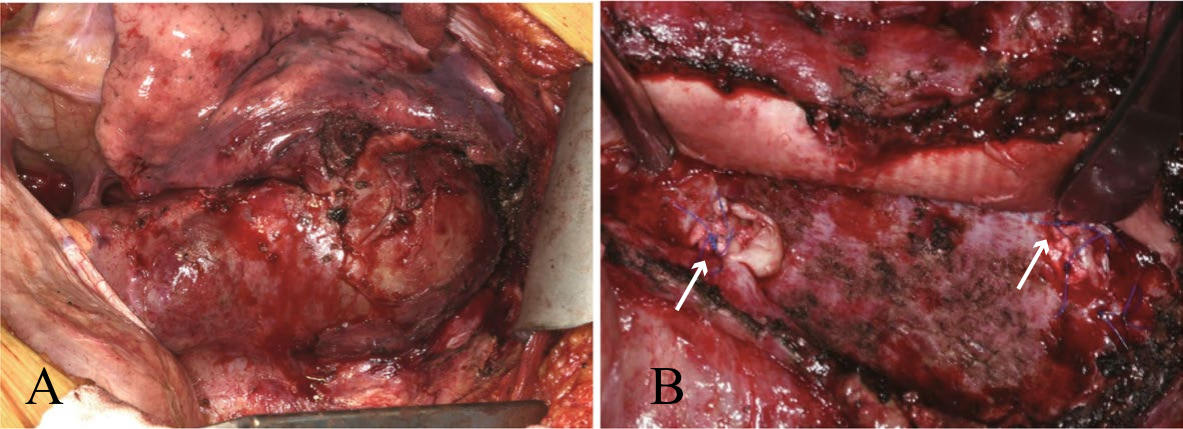
Intraoperative findings during hematoma removal from the native descending aortic wall and intercostal artery closure. **A** Aneurysm around the descending prosthetic graft. **B** White arrows indicate closed orifices of the right eighth and left tenth intercostal arteries

The patient was hospitalized and underwent left thoracotomy without cardiopulmonary bypass. We did not confirm the Adamkiewicz artery preoperatively or monitor motor-evoked potentials during surgery because we estimated a low risk of paraplegia by controlling backflow through recanalized intercostal arteries. In previous surgeries, large intercostal and lumbar arteries were reconstructed for spinal circulation, including the 6th and 11th intercostal and 1st and 3rd lumbar arteries. The internal pressure of the sac was 10 mm Hg when the systolic blood pressure was 90 mm Hg. Longitudinal incision of the aneurysm revealed abundant fresh and old clots inside the sac. The orifices of the right eighth and left tenth intercostal arteries were patent with backflow of blood and were closed using nonabsorbable sutures (Fig. 2). The orifice of the right seventh intercostal artery was also detected, but there was no blood backflow. This artery was suture ligated to prevent future recanalization. We found that the orifice of the left 7th intercostal artery had already been ligated in a previous surgery, and no other orifices of the intercostal arteries were detected. After suture ligation of the three detectable intercostal arteries, the arterial pressure was increased to 120 mmHg, and complete hemostasis from the intercostal arteries was confirmed. The graft was wrapped with the native aneurysmal wall. The patient’s postoperative course was uneventful, and she was discharged on postoperative day 10. CT performed 1 year later showed no aneurysmal recurrence.

## Discussion and conclusions

We experienced this very rare case of recurrent aortic aneurysm at 11 years after graft replacement resulting from recanalization of the intercostal arteries of the remaining aortic wall. This recanalization was similar to a type II endoleak after endovascular treatment. No backflow was observed during the thoracoabdominal replacement surgery; hence, the intercostal arteries were not closed.

It would be interesting to discern what triggered the intercostal artery recanalization 11 years after the aortic replacement. Because the PT-INR was remarkably high 1 month before her back pain appeared, an excess dose of anticoagulants might have dissolved the clots in the intercostal arteries. Some previous reports described similar situations in an abdominal aortic aneurysm [1–3]. One described a case of delayed recanalization of the lumbar artery after open repair of an abdominal aortic aneurysm [1]. Another described the recanalization of previously thrombosed type II endoleak after endovascular repair for an abdominal aortic aneurysm [3]. Systemic thrombolysis was suspected to be the cause of the events in both reports. Moreover, a previous study reported that persistent blood flow within the excluded aneurysm sac was found in 2% of patients who underwent abdominal aortic repair with a retroperitoneal aortic bypass, and that patients receiving chronic anticoagulation or those with extensive collaterals appear to be at higher risk for persistent flow within the aneurysm sac [2].

From these perspectives, mechanical valve-associated excessive anticoagulation is considered a reasonable cause of recanalization of the intercostal arteries in our case, even at a remote time after the original aortic surgery. After most of the intercostal and lumbar arteries were sacrificed via staged total aortic replacement, the slow formation of the collateral network might have affected the recanalization of the intercostal arteries. Although infection can influence recanalization, there were no apparent pre- or intraoperative findings suggestive of an active or healed infection. Its direct association with Marfan syndrome is uncertain, but the fragility of the aortic wall may provoke its expansion, even by low-pressure oozing through the intercostal arteries. Then, patients with Marfan syndrome tend to be at the aforementioned risk of undergoing multiple aortic and valve surgeries with possible mechanical valves in their life.

We chose to perform open repair through a left thoracotomy because the patient had symptoms of back pain due to an aneurysm compressing the esophagus. Transcatheter intervention to occlude the responsible intercostal arteries could be a less invasive treatment option in asymptomatic cases in the hope that the aneurysmal hematoma would gradually dissolve [4]. Closing all orifices of the intercostal arteries during open aortic repair could prevent delayed reopening of the intercostal arteries after aortic repair. After encountering this case, we routinely changed the strategy of aortic repair to close every orifice of the intercostal arteries, regardless of blood backflow. Furthermore, wrapping the graft with the native aorta could prevent pseudoaneurysm formation or bleeding into the thoracic cavity. Lifelong periodical observation by CT is essential for the early detection of unexpected and rare complications even after total aortic graft replacement.

In conclusion, here, we described a case in which a descending aortic aneurysm recurred 11 years after a graft replacement due to recanalization of the intercostal arteries of the wrapping native aorta like a type II endoleak in a patient with Marfan syndrome. Our findings suggest that, to prevent this delayed adverse event, the closure of all orifices of the intercostal arteries during aortic repair, regardless of backflow, is necessary. Systemic anticoagulation therapy after aortic replacement may be a risk factor for recanalization. Thus, lifelong periodic CT observation is essential, even after graft replacement of the entire aorta.

## Data Availability

All data used in the current study are included in this published article.

## Abbreviations

CT: Computed tomography
PT-INR: Prothrombin time-international normalized ratio
